# Supplementary material for: A Toolbox Model of Evolution of Metabolic Pathways on Networks of Arbitrary Topology
Source: PLoS Comput Biol. 2011 May 19;7(5):e1001137. doi: 10.1371/journal.pcbi.1001137 (PMC3098196; doi:10.1371/journal.pcbi.1001137)
Supplement: Text S1 — Supplementary information. (0.37 MB DOC) [file pcbi.1001137.s001.doc]

### Calculation of the average
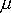
 in the toolbox model on a critical tree

The total fraction of metabolites from the universal network that are present in an organism specific network is given by

$\begin{matrix} \bar{\mu} & = & \sum_{d=1}^{d=d_{\max}} N_{M}\left(d\right)/\sum_{d=1}^{d=d_{\max}} N_{M}^{\left(U\right)}\left(d\right) \\ & = & \sum_{d=1}^{d=d_{\max}} \mu\left(d\right)N_{M}^{\left(U\right)}\left(d\right)/\sum_{d=1}^{d=d_{\max}} N_{M}^{\left(U\right)}\left(d\right) \end{matrix}$.

The boundary condition at the last layer of the tree does not satisfy the Eq. (4) but instead is given by$\mu\left(d_{\max}\right)=\tau$. One can easily show that for $d<d_{\max}$ $\mu\left(d\right)$rapidly (exponentially) converges to its steady state value $\mu=\sqrt{\tau}$and stays at this level for as long as $d\gg1$ when it starts rising again and ultimately approaches 1 at $d=0$. In a large critical network the number of nodes in the last and the first several layers is small compared to the total number of nodes in the network. Hence in case of a critical network one has$\bar{\mu}\approx\mu$

### Quadratic relation between
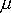
 and $\tau$ for general critical branching processes

The following equation relates $\mu\left(d\right)$ between consecutive layers on arbitrary critical branching process:

$$\mu\left(d\right)=\sum_{k=0}^{\infty} p_{k}\left\{1-\left[1-\mu\left(d+1\right)\right]^{k}\right\}+\tau p_{0}$$

Here $p_{k}$ is the probability for a node to branch out into *k* nodes, and the term $\left\{1-\left[1-\mu\left(d+1\right)\right]^{k}\right\}$ is the probability for a node that branched out into *k* nodes to have at least one of them picked. In the stationary state where $\mu$ is independent of *d*, we have

| $\begin{matrix} \mu& = & \sum_{k=0}^{\infty} p_{k}\left\{1-\left[1-\mu\right]^{k}\right\}+\tau p_{0} \\ & = & 1-\sum_{k=0}^{\infty} p_{k}\left[1-\mu\right]^{k}+\tau p_{0} \\ & = & \tau p_{0}+1-\sum_{k=0}^{\infty} \sum_{j=k}^{\infty} \left(-1\right)^{k+1}p_{j}C_{k}^{j}\mu^{k} \\ & = & \tau p_{0}+1-1+\mu\sum_{k=0}^{\infty} kp_{k}-\mu^{2}\sum_{k=0}^{\infty} \frac{k\left(k-1\right)}{2}p_{k}+O\left(\mu^{3}\right) \\ & & \end{matrix}$ |  |
| --- | --- |

A critical branching process has $\sum_{k=0}^{\infty} kp_{k}=1$, and this allows the first order term in the right hand side cancel with the $\mu$ on the left hand side, thus we have

| $\tau p_{0}=\mu^{2}\sum_{k=0}^{\infty} \frac{k\left(k-1\right)}{2}p_{k}+O\left(\mu^{3}\right)$ |  |
| --- | --- |

So finally we get $\tau\sim\mu^{2}+O\left(\mu^{3}\right)$ for$\mu,\tau\text{<<1}$, where the quadratic term dominates for small $\mu$.

Alternative derivation:

Another derivation, which is independent of layer-to-layer uniformity of branching probabilities *p_i_*, extends the proof from trees generated by Galton-Watson process to more general situations. This derivation starts with the conservation law describing changes of $N_{M}^{\left(U\right)}\left(d\right)$ in the universal network between two consecutive layers (this equation follows from the Eq. (1) in the manuscript when $\mu(d)=\tau=1$):

| $N_{M}^{\left(U\right)}\left(d\right)=N_{M}^{\left(U\right)}\left(d+1\right)-N_{B}^{\left(U\right)}\left(d\right)+N_{L}^{\left(U\right)}\left(d\right)$ |  |
| --- | --- |

For many trees the number of nodes in a layer changes slowly compared to the total number of nodes in a layer. For such trees $N_{M}^{\left(U\right)}\left(d+1\right)-N_{M}^{\left(U\right)}\left(d\right)$ is small compared with both $N_{B}^{\left(U\right)}\left(d\right)$ and $N_{L}^{\left(U\right)}\left(d\right)$ and thus one can approximately write $N_{B}^{\left(U\right)}\left(d\right)\cong N_{L}^{\left(U\right)}\left(d\right)$. Here as in the main text we assume that the universal network does not have more than 2 branches merging at a given node. In the stationary state where $\mu\left(d\right)\cong\mu\left(d+1\right)=\mu$ the Eq. (1) from the main text becomes

| $\mu N_{M}^{\left(U\right)}\left(d\right)=\mu N_{M}^{\left(U\right)}\left(d+1\right)-\mu^{2}N_{B}^{\left(U\right)}\left(d\right)+\tau N_{L}^{\left(U\right)}\left(d\right)$ and since $N_{B}^{\left(U\right)}\left(d\right)\cong N_{L}^{\left(U\right)}\left(d\right)$ and $N_{M}^{\left(U\right)}\left(d+1\right)\cong N_{M}^{\left(U\right)}\left(d\right)$ we once again get the quadratic scaling |  |
| --- | --- |

$\tau=\mu^{2}$ . This argument extends our proof of quadratic scaling to any tree-like universal network in which the number of nodes slowly changes from layer to layer.

### Solution to the toolbox model on a supercritical tree

$\mu\left(d\right)$ is the fraction of nodes in organism-specific network at distance $d$ from the origin of the tree satisfies the following difference equation:

| $\mu\left(d\right)=p\tau+k\mu\left(d+1\right)-\left(k-1+p\right)[\mu\left(d+1\right)]^{2}$ (S1) |  |
| --- | --- |

We are interested in small $\tau$ and so $\mu\left(d\right)$ is small, and by keeping only the leading linear term one gets $\mu\left(d\right)=p\tau+k\mu\left(d+1\right)$. The last layer is special since it contains only leaves and hence $\mu\left(d_{\max}\right)=\tau$ .

Iteratively solving Eq. (S1) one gets

| $\begin{matrix} \mu\left(d_{\max}-l\right) & = & p\tau+p\tau k+p\tau k^{2}+...+p\tau k^{l-1}+\tau k^{l} \\ & = & p\tau\left(k^{l}-1\right)/\left(k-1\right)+\tau k^{l} \\ & = & \tau k^{l}\left(k-1+p\right)/\left(k-1\right)-p\tau/\left(k-1\right) \\ & \approx& \tau k^{l}/\mu_{0} \end{matrix}$ | (6) |
| --- | --- |

where $\mu_{0}=\left(k-1\right)/\left(k-1+p\right)$. To arrive at this expression we have made an approximation by dropping the quadratic term in Eq. (S1). This made our estimation for $\mu\left(d\right)$ to increase without saturating at the steady state. To rescue this we assume that $\mu\left(d\right)$ follows the linearized difference equation until it reaches the steady state at the height$d_{\max}-m$, and then $\mu\left(d\right)$ stays as a constant over the region $d<d_{\max}-m$. Setting the equation $\tau k^{m}/\mu_{0}=\mu_{0}$ we will get

| $m=\log_{k}\left(\mu_{0}^{2}/\tau\right)$ | (7) |
| --- | --- |

Now we use the results of eq. (6) and (7) to calculate $n_{M}$, picking only the leading order:

| $\begin{matrix} N_{M} & = & \sum_{l=0}^{m} N_{M}^{\left(U\right)}\left(d_{\max}-l\right)\mu\left(d_{\max}-l\right)+\sum_{l=m+1}^{d_{\max}} N_{M}^{\left(U\right)}\left(d_{\max}-l\right)\mu\left(d_{\max}-l\right) \\ & = & k^{d_{\max}}\tau+\sum_{l=1}^{m} \left(k^{d_{\max}}/k^{l}\right)\left(\tau k^{l}/\mu_{0}\right)+\sum_{l=m+1}^{d_{\max}} \mu_{0}k^{d_{\max}}/k^{l} \\ & = & k^{d_{\max}}\tau+\left(\tau k^{d_{\max}}/\mu_{0}\right)m+\mu_{0}k^{d_{\max}}\sum_{l=m+1}^{d_{\max}} 1/k^{l} \\ & \approx& \left(\tau k^{d_{\max}}/\mu_{0}\right)\log_{k}\left(\mu_{0}^{2}/\tau\right)\approx N_{M}^{\left(U\right)}\frac{\tau}{\mu_{0}}\frac{k-1}{k}\log_{k}\left(\frac{\mu_{0}^{2}}{\tau}\right) \end{matrix}$ |  |
| --- | --- |

Finally we get $\mu=N_{M}/N_{M}^{\left(U\right)}=\frac{\tau}{\mu_{0}}\frac{k-1}{k}\log_{k}\left(\frac{\mu_{0}^{2}}{\tau}\right)$, where $\mu$scales with $\tau\log_{k}\left(\tau\right)$. The $\tau\log_{k}\left(\tau\right)$term comes from the summation of $\sum_{l=1}^{m} N_{M}^{\left(U\right)}\left(d_{\max}-l\right)\mu\left(d_{\max}-l\right)$, which represents the contribution from the last few layers before the saturation of $\mu\left(d\right)$.

### Error analysis of the toolbox model

The regression of the data of Figure 2 (*N_L_* vs. *N_M_* plot of the toolbox model on critical trees) and Figure 5 (*N_L_* vs. *N_M_* plot of the toolbox model on the metabolic network with branched pathways and multi-substrate reactions) in the manuscript was done first by logarithmically binning the data points along their *y*-coordinate (*N_L_*), and the exponents was then calculated with ordinary least square using the binned curve and taking the *y*-coordinate as the predictor and minimizing the mean square of the difference between the x-coordinate (log *N_M_* in our case) of the binned data and the fitted curve. The best fit coefficients of the linear regression and their 95% confidence intervals were calculated by the “regress” function of the Statistical Toolbox in Matlab 7. We used the *y*-coordinate as the predictor (as opposed to a more traditional use of the x-coordinate as the predictor) because in our simulations *N_L_* was increased in constant (unit) steps (one leaf was added per each step of the model), while the corresponding steps in *N_M_* (added pathways’ lengths) varied from simulation to simulation. Thus it was natural to view *N_M_* as a fluctuating function of *N_L_* and not vice versa.

The error bars on slopes (exponents) and prefactors in best linear fits to the binned data in Figure 2 and Figure 5 are based on the 95% confidence intervals estimated by the regress function in Matlab. Figure S1a shows that different fitting methods give consistent results, which indicates that the spread of the raw data is relatively small and will not change our main conclusions obtained from Figure 2 and Figure 5.


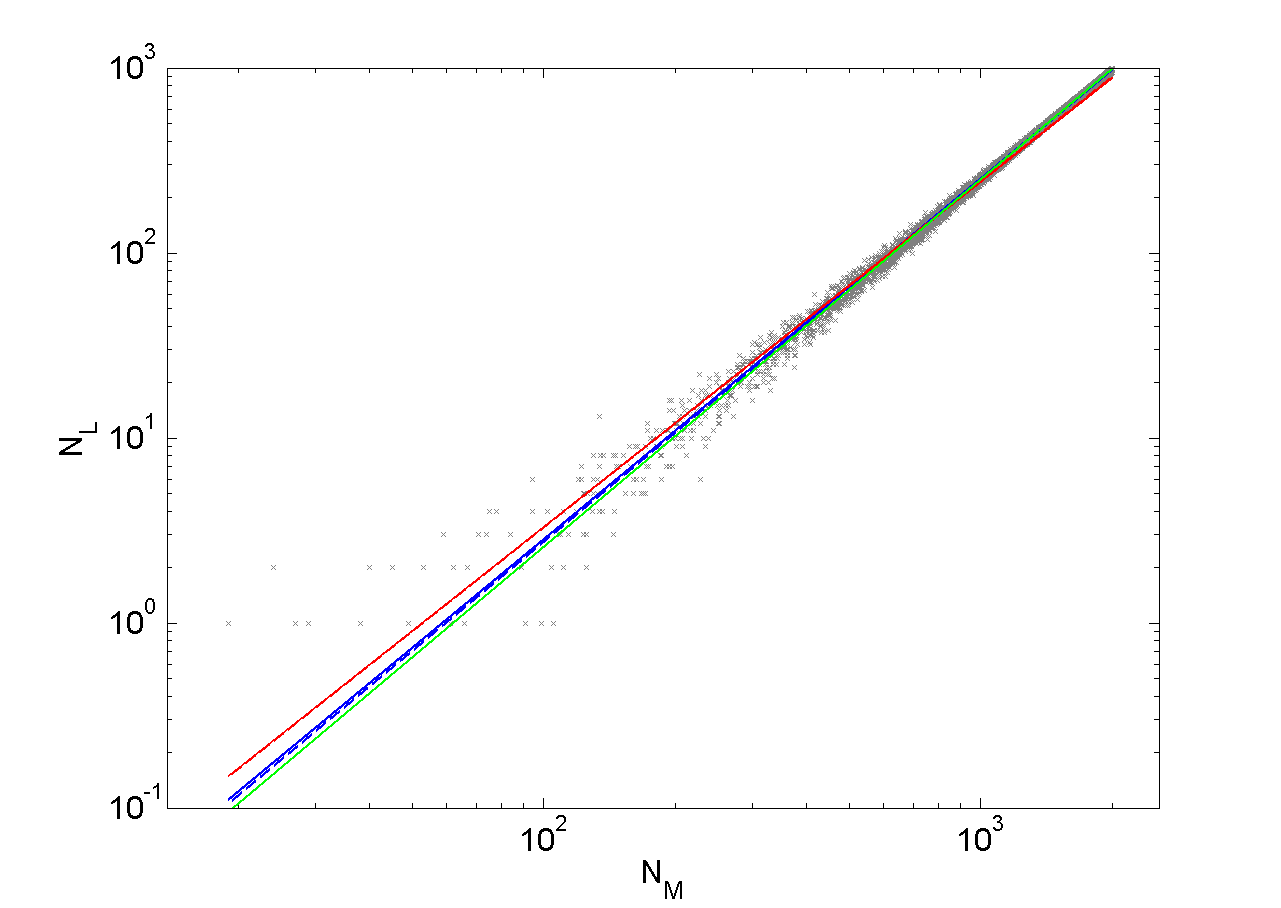


*Figure S1a Comparison of different ways of performing the regression analysis.*

$N_{L}$ *vs.* $N_{M}$ *data of the toolbox model on 10 different critical trees generated with probability p_0_ = 0.5 and p_2_ = 0.5. The grey crosses represents the raw data; the blue solid line is the best fit curve derived from linear regression, i.e., by taking the x-coordinate as the predictor and minimizing the mean square difference in y-coordinate between the best fit line and the data points in logarithmic scale; the blue dash line is the best fit curve derived from linear regression taking the y-coordinate as the predictor; the red solid line is the best fit curve obtained by first logarithmically binning the data along the y-coordinate followed by linear regression on the binned curve using the x-coordinate as the predictor in logarithmic scale; the red dash line is the best fit curve obtained by first logarithmically binning the data with their y-coordinate followed by linear regression on the binned curve using the y-coordinate as the predictor in logarithmic scale; the green solid line is the best fit curve obtained from powerlaw fitting of the raw data; the green solid curve is obtained by binning the along the y-coordinate and followed by fitting with powerlaw along the binned curve. All these best fit curves have exponent 1.9.*

Furthermore, as shown in Figure S1b, the exponent varies when we consider different range of *N_L_* in the regression analysis, and this indicates the existence of systematic error. The presence of the systematic error suggests that we cannot use the conventional regression analysis, because the error of the regression coefficients inversely depends on the square root of the number of data points. In our case we want the error to account for the systematic differences of exponent, and therefore we binned the data followed by regression. The binning of the data reduces the number of points and so the regression analysis that follows can reflect the change of the exponent along different regions, i.e., the systematic error, and get rid of the size effect of the size raw data.


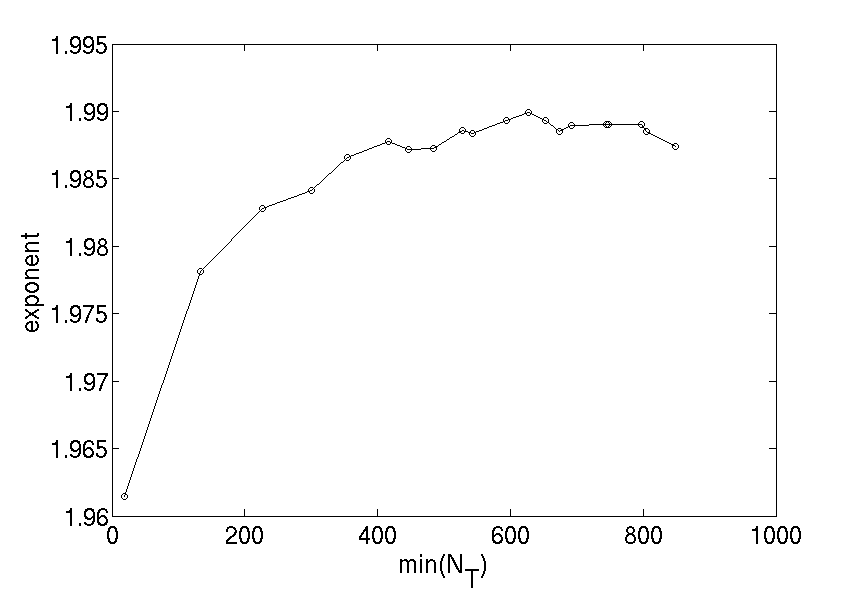


*Figure S1b Exponent obtained from regression on different ranges of N_L_.*

*The exponent is obtained by binning of data along the y-coordinates (N_L_) followed by regression using the x-coordinate as the predictor (N_M_) in logarithmic scale. The regression analyses are performed on different ranges of N_L_, where all share the same upper limit but their lower limits have different cutoffs.*

### Analysis of number of by-product of the pathways of the toolbox model on the metabolic network with branched pathways and multi-substrates reactions

Figure 7 of the manuscript shows the binned curve of *n_byproduct_* vs. *n_M_* but does not show any error or raw data. The raw data of the plot is shown in Figure S3, and the binning was done by first logarithmically grouping the data points according to their *x*-coordinate (*n_M_*) followed by arithmetic averaging of the *y*-coordinates of the data points (*n_byproduct_*). Geometric averaging on the *y*-coordinate was not used despite that the *x-*coordinate was binned logarithmically, because the majority of their values is 0 or 1.


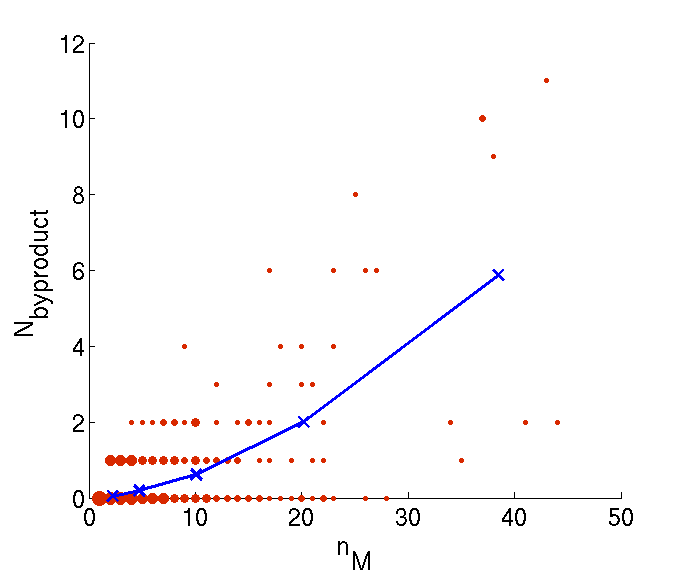


*Figure S2* $n_{bzproduct}$ vs. $n_{M}$*of the toolbox model in the metabolic network with branched pathways and multi-substrate reactions. Red circles: raw data; the scale of the circles is proportional to the logarithmic of its occurrence. Blue circles and dash line: binning of data by partitioning the x-axis logarithmically and average over y coordinates arithmetically.*

### Seed metabolites of the scope expansion model

The following 40 metabolites, except H2O, ATP and NAD, belong to the KEGG modules of central carbohydrate metabolism. They all serve as the starting core metabolites in the simulations of scope expansion.

Table S1. Seed metabolites of the scope expansion model

| **KEGG Entry number** | **Name** |
| --- | --- |
| C00001 | H2O |
| C00002 | ATP |
| C00003 | NAD+ |
| C00022 | pyruvate |
| C00024 | Acetyl-CoA |
| C00026 | 2-Oxoglutarate |
| C00036 | Oxaloacetate |
| C00042 | Succinate |
| C00074 | Phosphoenolpyruvate |
| C00085 | D-Fructose 6-phosphate |
| C00091 | Succinyl-CoA |
| C00111 | Glycerone phosphate |
| C00117 | D-Ribose 5-phosphate |
| C00118 | D-Glyceraldehyde 3-phosphate |
| C00119 | 5-Phospho-alpha-D-ribose 1-diphosphate |
| C00122 | Fumarate |
| C00149 | L-Malate |
| C00158 | Citrate |
| C00197 | 3-Phospho-D-glycerate |
| C00199 | D-Ribulose 5-phosphate |
| C00204 | 2-Dehydro-3-deoxy-D-gluconate |
| C00231 | D-Xylulose 5-phosphate |
| C00236 | 3-Phospho-D-glyceroyl phosphate |
| C00257 | D-Gluconate |
| C00267 | alpha-D-Glucose |
| C00279 | D-Erythrose 4-phosphate |
| C00311 | Isocitrate |
| C00345 | 6-Phospho-D-gluconate |
| C00577 | D-Glyceraldehyde |
| C00631 | 2-Phospho-D-glycerate |
| C00668 | alpha-D-Glucose 6-phosphate |
| C01172 | beta-D-Glucose 6-phosphate |
| C01236 | D-Glucono-1,5-lactone 6-phosphate |
| C04442 | 2-Dehydro-3-deoxy-6-phospho-D-gluconate |
| C05345 | beta-D-Fructose 6-phosphate |
| C05378 | beta-D-Fructose 1,6-bisphosphate |
| C05382 | Sedoheptulose 7-phosphate |
| C15972 | Lipoamide-E |
| C15973 | Dihydrolipoamide-E |
| C16254 | S-Succinyldihydrolipoamide-E |

### Alternative model: each organism evolves its own network in the absence of horizontal gene transfers

In the toolbox model where the organisms evolve mainly by HGT, the union of all metabolic enzymes forms the universal network which can be represented by a critical tree. One might wonder if the $N_{L}$ vs. $N_{M}$ plot will remain quadratic if we turn off the HGT and assume that de novo formation of new protein is the only way to evolve, and the metabolic network of each organism is an independent tree. To study this alternative model, we repeated the Galton-Walton process and generate a set of critical trees, and consider the $N_{L}$ vs. $N_{M}$ plot. Theoretically, the $N_{L}$ vs. $N_{M}$ plot of this alternative model will no longer demonstrate any quadratic scaling but only a linear one, with slope being $p_{0}$, the probability for a node to terminate in a branching process. The simulation of the alternative model was performed by repeating the branching process, and that *N_L_ = p_0_ N_M_* as verified (see Figure S3).


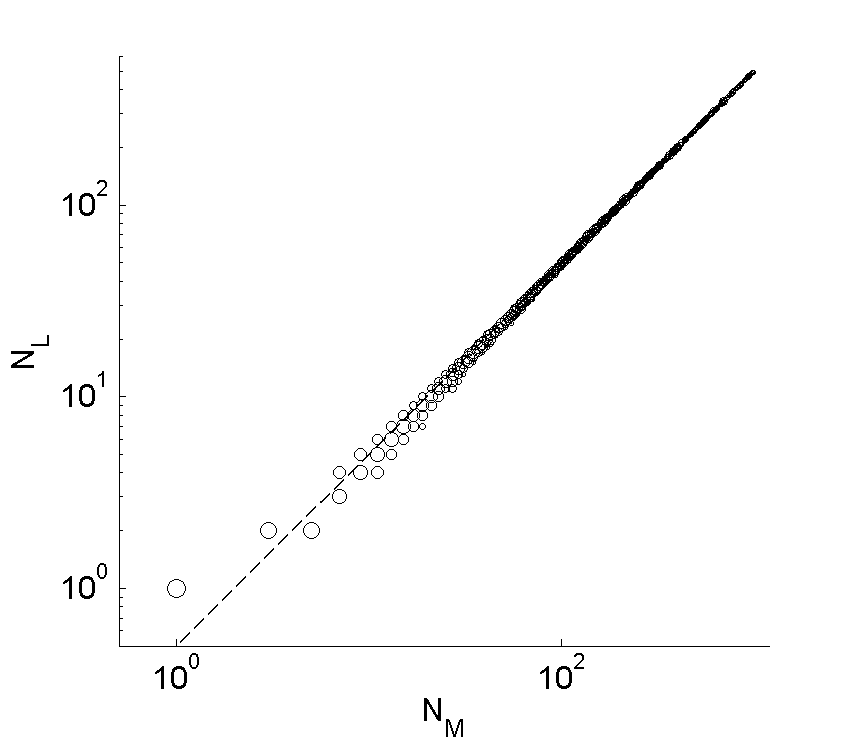


*Figure S3* $N_{L}$ vs. $N_{M}$*of trees. The trees are generated with Galton-Watson process with probability 0.5 to terminate and 0.5 to branch out into 2 children. Scale of circles is proportional to the logarithm of the density of data and the dash line is the theoretical prediction, which is* $N_{L}$=$p_{0}N_{M}$

### Analysis of currency metabolites in the toolbox model

The currency metabolites, i.e., metabolites that serve as inputs for a large number of pathways, have special roles in the metabolic network. In the analysis of the border reactions of on the metabolic pathways of the toolbox model in the manuscript, we concluded, without taking into account of the currency metabolites, that the pathways of the toolbox model are all most linear and lie on the surface of the core, receiving metabolites from the core and feeding them back. One might wonder how the geometry of the pathways will be affected if we take into account the currency metabolites. To study their effects, first of all, let us define *l_i_* to be the number of times node *i* act as a substrate or feedback to a pathway in one simulation. The toolbox model simulation was repeated several times and we can get the average *l_i_*. Analysis of the data showed that the distribution of *l_i_* is divided into two groups (the two steep kinks in Figure S4a), and we picked the top ten metabolites with the largest average *l_i_* to be currency metabolites and plotted the *n_border rxn_* vs. *n_rxn_* (Figure S4b). The average number of currency metabolites that a typical pathway connects is around 1 (data no shown).


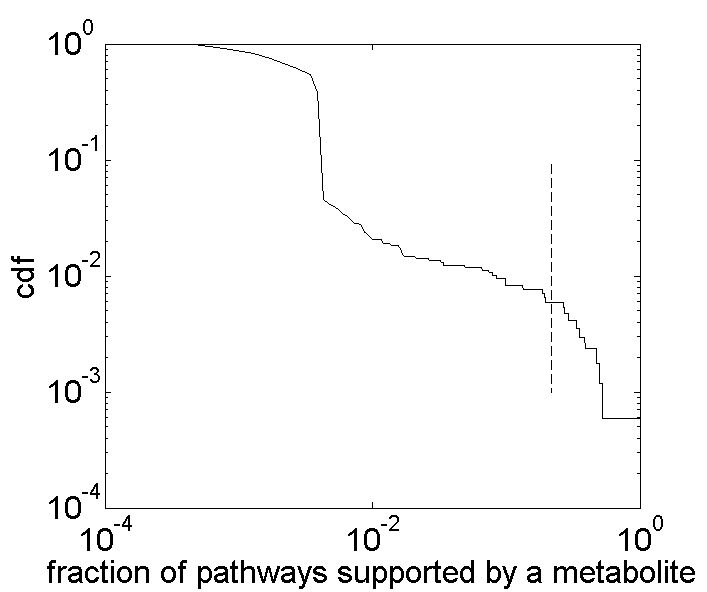


*Figure S4a Distribution of the fraction of pathways a metabolite supports. The dash line indicates the cutoff, where to the right of the dash line defines the currency metabolites.*


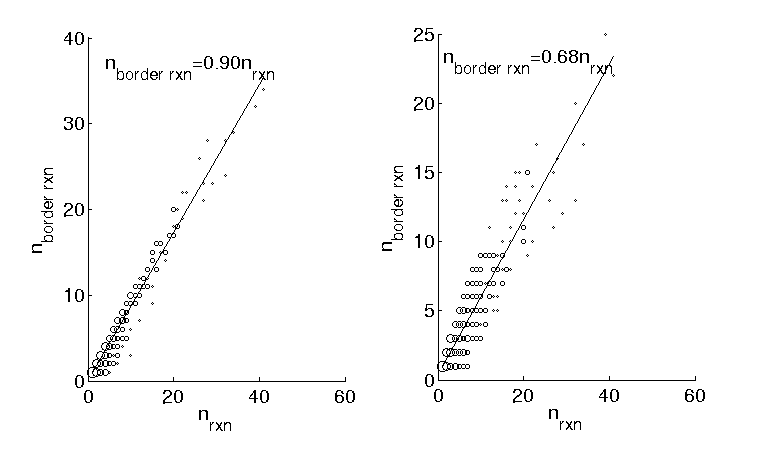


*Figure S4b n_border rxn_*  vs. n_rxn_ *before (left) and after (right) the removal of currency metabolites.*

The figures indicate that the removal of currency metabolites does affect the number of border reactions and reduce their number by a quarter. Nevertheless this does not change our conclusion that pathways are rather linear and lie on the surface of metabolic core.
